# Elevated Polyreactive Immunoglobulin G in Immune‐Mediated Liver Injuries With the Need for Immunosuppressive Therapy

**DOI:** 10.1111/liv.70571

**Published:** 2026-03-08

**Authors:** Theresa Kirchner, George N. Dalekos, Kalliopi Zachou, Mercedes Robles‐Díaz, Raúl J. Andrade, Marcial Sebode, Ansgar Lohse, Maciej K. Janik, Piotr Milkiewicz, Mirjam Kolev, Nasser Semmo, Tony Bruns, Tom Jg. Gevers, Benedetta Terziroli Beretta‐Piccoli, Heiner Wedemeyer, Elmar Jaeckel, Richard Taubert, Bastian Engel, pIgG study group

**Affiliations:** ^1^ Department of Gastroenterology, Hepatology, Infectious Diseases and Endocrinology Hannover Medical School Hannover Germany; ^2^ European Reference Network on Hepatological Diseases (ERN RARE‐LIVER) Hamburg Germany; ^3^ Department of Medicine and Research Laboratory of Internal Medicine, National Expertise Center of Greece in Autoimmune Liver Diseases General University Hospital of Larissa Larissa Greece; ^4^ Unidad de Gestión Clínica de Aparato Digestivo, Instituto de Investigación Biomédica de Málaga‐IBIMA, Plataforma BIONAND, CIBEREHD, Hospital Universitario Virgen de la Victoria Universidad de Málaga Málaga Spain; ^5^ Department of Medicine University Medical Centre Hamburg‐Eppendorf Hamburg Germany; ^6^ Department of Hepatology, Transplantology, Internal Medicine Medical University of Warsaw Warsaw Poland; ^7^ Translational Medicine Group Pomeranian Medical University Szczecin Poland; ^8^ Department of Visceral Surgery and Medicine, Inselspital, Bern University Hospital University of Bern Bern Switzerland; ^9^ Department of Medicine III University Hospital RWTH Aachen Aachen Germany; ^10^ Department of Gastroenterology and Hepatology Maastricht University Medical Center Maastricht the Netherlands; ^11^ Epatocentro Ticino Lugano Switzerland; ^12^ PRACTIS & CORE100Pilot Clinician Scientist Programs, Dean's Office for Academic Career Development Hannover Medical School Hannover Germany

**Keywords:** autoimmune hepatitis, drug induced autoimmune‐like hepatitis, drug induced liver injury, polyreactive immunoglobulin G

## Abstract

**Background & Aims:**

The distinction of drug‐induced liver injury (DILI), drug‐induced autoimmune‐like hepatitis (DI‐ALH), and autoimmune hepatitis (AIH) can be challenging due to overlapping clinical characteristics. Recently, polyreactive immunoglobulin G (pIgG) was identified as a novel biomarker in AIH. This retrospective study aimed to evaluate the diagnostic accuracy of pIgG to distinguish between AIH, DI‐ALH, and DILI and thus identify patients in need of immunosuppression.

**Methods:**

Samples from 120 patients (AIH = 81, DI‐ALH = 16, DILI = 23) were compared to a control group (non‐AIH‐non‐DILI‐liver disease = 596 and healthy controls = 190).

**Results:**

No patient in the DILI‐group but 98% in the AIH‐ and 94% in the DI‐ALH‐group received immunosuppressive treatment. PIgG levels were significantly higher in the AIH‐group 1.9 normalised arbitrary units (nAU) compared to DILI (1.1 nAU, *p* < 0.001), non‐AIH‐non‐DILI‐LD (1.0 nAU, *p* < 0.001) and healthy controls (0.27 nAU, *p* < 0.001). PIgG levels for DI‐ALH (1.7nAU) were significantly higher compared to DILI (*p* = 0.044) and non‐AIH‐non‐DILI‐LD and healthy controls (both *p* < 0.001). Highest AUC was seen for pIgG (0.818) compared to conventional autoantibodies. The overall accuracy of pIgG to distinguish AIH from DILI (74%) and liver injuries with and without the need for immunosuppression (73%) was like that of ANA (71%/73%) and SMA (74%/69%) at cut‐offs of ≥ 1/40. PIgG was positive in up to 79% of patients with AIH that were negative for a conventional autoantibody and was positive in 90% of DI‐ALH cases compared to 25% in DILI that were caused by the same drugs.

**Conclusions:**

PIgG may complement current serologic tests to identify patients with liver injury in need of immunosuppressive treatment.

## Lay Summary

The distinction between autoimmune liver diseases that require immunosuppressive treatment and liver diseases caused by medications or other toxic substances is often very difficult. Polyreactive immunoglobulin G has been found to be elevated in patients with autoimmune hepatitis. This study was able to demonstrate that polyreactive immunoglobulin G can also be used to differentiate between autoimmune liver diseases that require immunosuppressive therapy and toxic liver diseases.

**Abbreviations**
AIH: autoimmune hepatitis
aLKM: anti‐liver kidney microsomal antibodies
ANA: antinuclear antibody
aSLA: anti‐soluble liver antigens
aSMA: anti‐smooth‐muscle antigen
DI‐ALH: drug‐induced autoimmune‐like hepatitis
DILI: drug‐induced liver injury
IFT: immunofluorescence testing
liver disease: non‐AIH‐non‐DILI‐LD non‐autoimmune hepatitis non‐drug‐induced liver injury
pIgG: polyreactive immunoglobulin G

## 1 Introduction

Drug‐induced liver injury (DILI) and autoimmune hepatitis (AIH) are difficult to differentiate from each other as they often share various clinical characteristics [1]. AIH is a rare immune‐mediated disease that can lead to acute liver failure or cirrhosis and liver‐related death when untreated, but when treated correctly prognosis is good in most of the cases [2]. The diagnosis is based on autoantibody‐measurements, histopathological findings and elevation of immunoglobulin G (IgG) [3]. Continuous immunosuppressive therapy is needed to treat AIH and to prevent the progression of liver disease [3]. Although there is a diagnostic score AIH remains a diagnosis of exclusion because there is no specific diagnostic marker [4].

Drug‐induced liver injury (DILI) can mimic the majority of other liver disorders and is triggered by various causative agents, for example, antibiotics, metamizole, psychotropic drugs, or herbal medication and dietary supplements [5, 6]. Clinical presentation ranges from asymptomatic elevation of liver enzymes to acute liver failure [5]. Idiosyncratic DILI affects only susceptible individuals and is less related to the drug dose [7]. A temporal relationship with the suspected causative agent is needed and other competing etiologies have to be ruled out; however, there is no specific diagnostic tool for DILI [5]. Autoimmune features are rarely seen in DILI [8]. After discontinuation of the causative agent, DILI is a self‐limiting disease with a good prognosis [5]. Complementary to typical DILI, another drug‐related liver disease with autoimmune features is increasingly reported in the last years: drug‐induced autoimmune‐like hepatitis (DI‐ALH). DI‐ALH is defined as a liver injury with laboratory and/or histological features that may be indistinguishable from idiopathic AIH [6]. Previous studies showed that histological, biochemical, and immunological features are overlapping in both immune‐mediated entities [6].

Patients with AIH require long‐term immunosuppressive therapy whereas immunosuppressive therapy can be safely withdrawn after weeks or a few months in DI‐ALH [9]. As distinction between AIH and DI‐ALH is impossible in most cases at baseline based on the currently available diagnostic tools, immunosuppressive therapy is often started pragmatically and the final diagnosis can only be confirmed by the success or failure of an immunosuppression withdrawal attempt during follow‐up [6].

Autoantibody measurement is a cornerstone in the non‐invasive diagnostic work‐up of any unclear hepatitis. The most frequent conventional autoantibodies are antinuclear antibodies (ANA) and anti‐smooth‐muscle antigen (aSMA) antibodies in AIH type 1, while anti‐liver‐kidney‐microsomal (aLKM) and/or liver‐cytosolic type 1 antibodies define AIH type 2. However, there is either a lack in sensitivity or in specificity for AIH, causing diagnostic uncertainty [10, 11, 12].

Polyreactivity describes the potential of an autoantibody to bind multiple molecular structures; this leads to a higher neutralising potential. Polyreactive immunoglobulins play an important role in primary immune response and in apoptosis. In 2022, polyreactive IgG (pIgG) was identified as a promising new biomarker to improve the diagnostic workup of any non‐viral hepatitis with higher specificity and overall accuracy to distinguish AIH from non‐AIH liver diseases than conventional autoantibodies in adults [13] and children [14] with additional value in autoantibody‐negative AIH. However, DI‐ALH and DILI were underrepresented in this initial characterisation of pIgG [13].

This retrospective multicenter study aims to evaluate the diagnostic capacity of pIgG to predict AIH in comparison to DI‐ALH, DILI, and non‐AIH‐non‐DILI liver diseases (non‐AIH‐non‐DILI‐LD). The distinction is clinically important to withhold immunosuppressive treatment in patients with DILI that do not need such treatment and differentiate AIH from DI‐ALH to identify patients in whom immunosuppressive treatment may be safely discontinued.

## 2 Patients and Methods

### 2.1 Definitions

#### 2.1.1 AIH

In accordance with clinical guidelines the simplified AIH score was ≥ 6 (3) and the diagnosis was biopsy proven in every case. Clinical diagnosis and disease courses were compatible with AIH and patients were dependent on immunosuppressive therapy longer than 6 months after diagnosis [3].

#### 2.1.2 Di‐ALH

DI‐ALH was diagnosed in accordance with the expert opinion published by Andrade et al. in 2023 [6]. For DI‐ALH there was at least one suspected causative agent, and laboratory and histological features were compatible with DI‐ALH according to the recently published criteria. Immunosuppressive therapy was initiated for remission induction in 94% of the cases, but there was no dependency on immunosuppressive medication for more than 6 months after the diagnosis [6]. There was no relapse after cessation of immunosuppressive therapy till the end of the study in all cases.

#### 2.1.3 Dili

Definition was based on the latest EASL guidelines for DILI [5]. DILI was characterised by the existence of at least one suspected causative agent and the clinical and histological features as well as the disease course judged by the treating physicians were compatible with DILI. There was no need for immunosuppressive therapy at any time from diagnosis to last follow‐up.

### 2.2 Study Population

One hundred twenty adult patients (age ≥ 18 years) with AIH (*n* = 81), DI‐ALH (*n* = 16), and DILI (*n* = 23) without pre‐existing liver disease from nine European centers were newly recruited for this retrospective multicenter study from existing biorepositories. Study groups were compared to an already established non‐AIH‐non‐DILI‐liver disease (non‐AIH‐non‐DILI‐LD) control group and to healthy controls (*n* = 190) from a previous study [13]. The non‐AIH‐non‐DILI‐LD group (*n* = 596) particularly included non‐alcoholic fatty liver disease (*n* = 204), primary sclerosing cholangitis (*n* = 147), primary biliary cholangitis (*n* = 125), and alcoholic liver disease (*n* = 90).

All included patients with AIH, DI‐ALH, non‐AILD‐non‐DILI‐LD and 61% of patients with DILI underwent a diagnostic liver biopsy for the work‐up of unclear non‐viral acute hepatitis. Serum samples were stored directly after onset and before the beginning of immunosuppressive treatment in every case. Samples were stored between 1990 and 2023. Patients with a loss to follow up within the first 6 months after liver biopsy or on immunosuppressive therapy prior to the liver biopsy were excluded.

### 2.3 Quantification of Polyreactive Immunoglobulin G

Samples were stored at ≤ −20°C at Hanover Medical School. Samples from the other centers were cryo‐conserved locally and sent frozen to Hanover. Quantification of pIgG was done using a custom‐made Enzyme‐linked Immunosorbent Assay (ELISA) with reactivity against human huntingtin‐interacting protein 1‐related protein (autoantigen) in bovine serum albumin blocked ELISA (HIP1R/BSA) in a single 1:100 dilution as published recently [13, 14, 15]. A standard curve was computed from five reference samples to calculate arbitrary units (AU) for each sample from the standard curve. AUs were normalised for center background and storage duration as published (normalised AU: nAU) [13]. The test is outlined in more detail in the Supporting Information. Autoantibody measurement was performed according to local standards in the participating centers in accordance with clinical guidelines [3, 16].

### 2.4 Ethics

Written informed consent was obtained from all patients at respective centers. Use of material and data from all patients in this multicenter study was approved by the respective local ethical committees. The study was approved by the local Ethics Committee (Number 5582 with last Update from 2018, Hannover Medical School Ethics Committee, Hannover Medical School, Hannover, Germany). The study conforms to the ethical guidelines of the 1975 Declaration of Helsinki.

### 2.5 Statistical Analysis

Statistical analysis was performed using SPSS (version 15.0, SOSS; Inc., Chicago, IL), GraphPad Prism (version 10; GraphPad Prism Software Inc., La Jolla, CA), and Microsoft Excel (version 2019, Redmond, Washington).

Categorical variables are expressed as numbers and percentages; continuous variables are expressed as median and range. Chi^2^‐test was used to compare contingency tables. The Mann–Whitney *U* test was used to compare quantitative data between two groups, and the Kruskal–Wallis test was used to compare quantitative data between more than two groups. Area under the receiver operating characteristic (AUC) analyses and Youden's index were used to identify cut‐off values. Accuracy of the diagnostic test was calculated as: (true positive + true negative)/total number. Sensitivities and specificities were compared with McNemar's test.

*p*‐values < 0.05 (two‐tailed) were considered significant in all analyses.

## 3 Results

### 3.1 Patient Characteristics

This multicenter analysis included 81 patients with AIH, 16 with DI‐ALH and 23 with DILI that were newly recruited for this study, as well as a previously published comparator cohort of 596 patients with non‐AIH‐non‐DILI‐LD and 190 healthy controls taken from our previous study [13]. Table 1 summarises the main demographic and laboratory features of the study population. The median age was lowest in healthy controls and highest in the AIH‐group (*p* = 0.002). The proportion of females was significantly higher in immune mediated liver diseases (AIH 79%, DI‐ALH 75%) compared to DILI (57%), non‐AIH‐non‐DILI‐LD (55%) and healthy controls (35%, *p* < 0.001). AST and ALT were highest in DI‐ALH and lowest in non‐AIH‐non‐DILI‐LD (both *p* < 0.001). IgG elevation in AIH (1.06 xULN) was higher compared to DI‐ALH (0.89 xULN), DILI (0.75 xULN) and non‐AIH‐non‐DILI‐LD (0.80 xULN, *p* < 0.001, Table 1). IgG correlated weakly to moderately with pIgG (Spearman‐Rho 0.62, *p* < 0.001), ANA (Spearman‐Rho 0.281, *p* = 0.012) and aSMA (Spearman‐Rho 0.312, *p* = 0.013) in patients with AIH. IgG correlated also with ANA in patients with DI‐ALH (Spearman‐Rho 0.661, *p* = 0.007) and with pIgG and ANA in patients with DILI (Spearman‐Rho 0.424 and 0.536, *p*‐values 0.049 and 0.012).

**TABLE 1:** Baseline characteristics.

	AIH (*n* = 81)	DI‐ALH (*n* = 16)	DILI (*n* = 23)	Non‐AIH‐non‐DILI‐LD (*n* = 596)	HC (*n* = 190)	*p*
Age (years)	53 (18; 78)	46 (24; 81)	50 (23; 77)	49 (18; 83)	44 (19;88)	0.002
Female sex	64 (79)	12 (75)	13 (57)	327 (55)	66 (35)	< 0.001
ALT [xULN]	16.62 (1.03; 90.65)	41.05 (3.88; 91.18)	8.65 (1.30; 91.76)	1.22 (0.20; 125.36)		< 0.001
AST [xULN]	14.23 (0.7; 115.0)	38.98 (3.16; 92.51)	4.50 (0.65; 79.71)	1.0 (0.02; 46.37)		< 0.001
AP [xULN]	1.11 (0.43; 10.40)	1.82 (0.59; 33.07)	1.96 (0.49; 12.41)	1.21 (0.34; 10.91)		0.018
gGT [xULN]	3.92 (0.67; 41.47)	4.86 (0.91; 38.00)	4.61 (0.58; 36.0)	2.18 (0.24; 47.03)		< 0.001
Bilirubin [xULN]	2.05 (0.24;22.83)	8.44 (0.83; 34.43)	1.10 (0.17; 29.67)	0.67 (0.15; 37.33)		< 0.001
IgG [xULN]	1.06 (0.15;2.92)	0.89 (0.44; 2.14)	0.75 (0.42; 1.55)	0.80 (0.09; 2.38)		< 0.001
IgG elevation, yes	47 (59)	2 (13)	4 (18)	97 (28)		< 0.001
pIgG (nAU)	1.87 (0.43; 5.85)	1.66 (0.74; 3.28)	1.12 (0.31; 1.99)	1.00 (0.15; 3.46)		< 0.001

*Note:* Categorical variables are expressed as numbers and percentages (*p*‐value is qui‐square); continuous variables are expressed as median and range (*p*‐value is Kruskal–Wallis).

Abbreviations: ULN: upper limit of normal, nAU: normalised arbitrary units.

Suspected causative agents of DILI and DI‐ALH are shown in Table 2. Antibiotics and metamizole were the causative agents in most cases. Because of polypharmacy, there was more than one drug that potentially triggered the disease in 17% of patients with DILI and in 25% of patients with DI‐ALH.

**TABLE 2:** Suspected causative agents in DILI and DI‐ALH.

Suspected causative agent	DILI		DI‐ALH	
Multiple agent^a^	*n* = 5		*n* = 5	
Antibiotics	*n* = 4	Minocyclin, cefazoline, ciprofloxacin		
Metamizol	*n* = 3		*n* = 5	
Methylprednisolone	*n* = 1		*n* = 2	
Protein kinase inhibitors	*n* = 1	Cobimetinib		
Monoclonal antibodies	*n* = 1	Natalizumab		
Muscle relaxant	*n* = 1	Tizanidine		
Antihypertensive drugs	*n* = 1	Dihydralazine		
Interferone	*n* = 1			
Hydroxychloroquine	*n* = 1			
Antidepressant	*n* = 1	Fluoxetin	*n* = 1	Opipramol
mRNA based SARS‐CoV‐2 vaccine	*n* = 3		*n* = 2	
Mesalazine			*n* = 1	

^a^ Patients took several medications with more than one possible causative agent.

### 3.2 Immunosuppressive Treatment in AIH, DI‐ALH and DILI

Ninety‐eight percent of patients (*n* = 79) in the AIH‐group were treated with immunosuppressive therapy for remission induction (Table S1). One patient refused to take the recommended medication, and another had spontaneous remission without any therapy. One patient in the DI‐ALH group refused to take the recommended immunosuppressive medication; therefore, 94% of the DI‐ALH group received immunosuppressive medication for remission induction. According to the given definitions, no patient in the DILI‐group received immunosuppressive therapy. In most cases, corticosteroid monotherapy (51%) was initiated, followed by a combination of steroids and mycophenolate mofetil in 28% in the AIH‐group. At month six, 98% of patients in the AIH‐group were treated with immunosuppressive therapy, while no patient in the DI‐ALH or DILI groups had immunosuppressive treatment after 6 months. Immunosuppressive therapy changed from steroid monotherapy for remission induction to combination with MMF or azathioprine in most of the cases in the AIH‐group. Comparative patient data are summarised in Table S1.

### 3.3 Polyreactive IgG Was Significantly Higher in Mmune‐Mediated Liver Disease (AIH and DI‐ALH)

**FIGURE 1:**
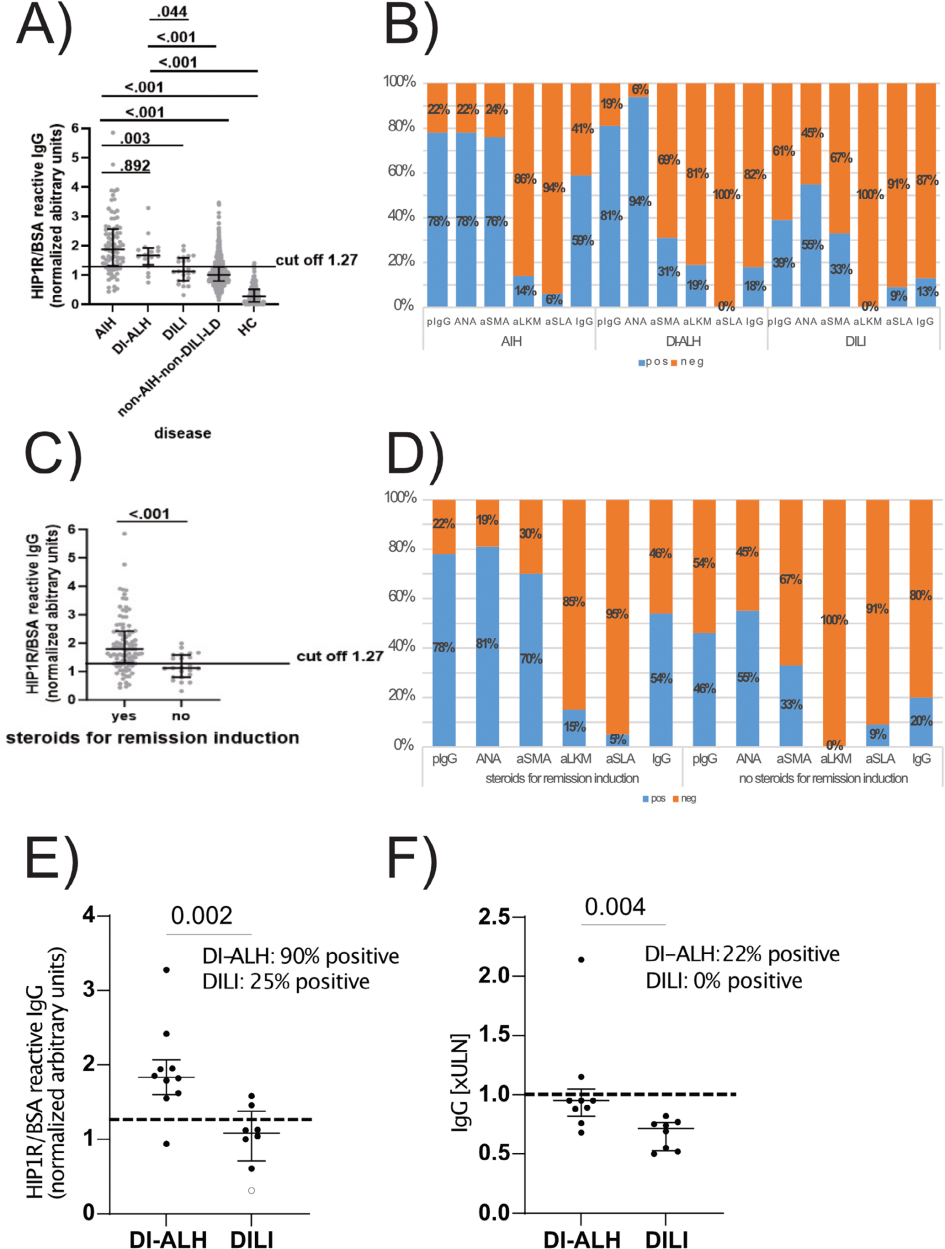
Polyreactive immunoglobulin G in comparison to conventional autoantibodies and IgG for the identification of AIH and immunosuppressive‐dependent liver injury. Concentration of pIgG, expressed as nAU, in the different groups (autoimmune hepatitis (AIH), drug‐induced autoimmune‐like hepatitis (DI‐ALH), drug‐induced liver injury (DILI), non‐AIH‐non‐DILI‐liver diseases (non‐AIH‐non‐DILI‐LD) and healthy controls (HC)) (A) and in patients with and without immunosuppressive therapy for remission induction (C). The cut‐off of 1.27 nAU was recently published for the distinction between untreated AIH and non‐AIH liver diseases. Positivity and titre for conventional autoantibodies in the different groups (B) and in patients with and without immunosuppressive therapy for remission induction (D). Distribution of pIgG values (HIP1R/BSA reactive IgG) (E) and IgG (F) in patients with either DI‐ALH or DILI caused by the same drugs. aLKM, Anti‐liver kidney microsomal antibodies; ANA, Antinuclear antibodies; aSLA, Anti‐soluble liver antigen antibodies; aSMA, Anti‐smooth muscle antigen antibodies; BSA, Bovine serum albumin; HIP1R, Huntingtin‐interacting protein 1‐related protein; IgG, Immunoglobulin G; nAU, Normalised arbitrary units; PIgG, Polyreactive immunoglobulin G; ULN, Upper limit of normal.

The median pIgG level was highest in the AIH‐group (1.87 nAU, range 0.43–5.85 nAU, Figure 1A) and significantly higher compared to the DILI‐ (1.12 nAU, range 0.31–1.99 nAU, *p* = 0.003) and the non‐AIH‐non‐DILI‐LD‐group (1.00 nAU, range 0.15–3.46 nAU, *p* < 0.001). The median pIgG level in the DI‐ALH group (1.66 nAU, range 0.74–3.28 nAU) was significantly higher compared to DILI (*p* = 0.044) and non‐AIH‐non‐DILI‐LD (*p* < 0.001). Lowest median pIgG level was seen in healthy controls (0.27 nAU, range 0.00–1.40, Figure 1A). Aside from that, the difference between AIH and DI‐ALH (*p* = 0.892) and between DILI and non‐AIH‐non‐DILI‐LD (*p* = 0.274) was not significant. When applying the previously established cut‐off 1.27 nAU [13] 78% of AIH, 81% of DI‐ALH and 39% of DILI patients were pIgG positive whereas IgG was elevated in 59%, 18% and 13% respectively (Figure 1B).

When comparing all cases with immunosuppressive therapy for remission induction in the AIH‐ and the DI‐ALH‐group (*n* = 94) to those liver injuries without immunosuppressive therapy for remission induction (DILI group, *n* = 23) pIgG levels were significantly higher in patients with the need for immunosuppressive therapy (1.80 nAU (range 0.43–5.85 nAU) vs. 1.12 nAU (range 0.31–1.99 nAU), *p* < 0.001, Figure 1C). When applying the previously established cut‐off 1.27 nAU [13] 78% of patients with subsequent steroid medication were pIgG‐positive, while only 46% of patients that were not treated with immunosuppressive therapy were positive for pIgG. In parallel, 54% and 20% of patients that were treated with and without immunosuppression had elevated IgG (Figure 1D).

Some drugs were implicated as causative agents in both DI‐ALH and DILI patients (Table 2). In cases caused by the same agents, pIgG was positive in 90% of DI‐ALH cases, compared to 25% of DILI cases (Figure 1E). Conversely, IgG (cut‐off > ULN) was positive in 22% of DI‐ALH cases but negative in all DILI cases (Figure 1F).

When conventional autoantibodies were negative or IgG was normal, pIgG was positive in up to 79% of patients with AIH and 100% of patients with DI‐ALH, but only 40% of patients with DILI (see Figure S1A–E).

### 3.4 Autoantibody Measurement and Serological Features in AIH, DI‐ALH and DILI

Positive serology (any positivity for ANA/aSMA/aLKM ≥ 1/40 and/or positivity for aSLA) was significantly more frequent in AIH (96%) and DI‐ALH (92%) compared to DILI (70%, *p* = 0.001).

The frequency of any antibody positivity was comparable between AIH and DI‐ALH (*p* = 0.683).

Positivity for ANA was highest in the DI‐ALH group (94%) compared to the AIH‐(78%) and the DILI‐group (55%, *p* = 0.106, Figure S2A). Titers for ANA were significantly higher in the AIH‐ and DI‐ALH groups (*p* < 0.001). Staining pattern was fine speckled (AIH and DI‐ALH both 46%), homogenous (AIH 23%, DI‐ALH 13%) and nucleolar (AIH 5%, DI‐ALH 34%) in most of the immune‐mediated cases. Highest positivity for aSMA was seen in the AIH‐group (76%, *p* < 0.001). In addition, there were higher aSMA titers in AIH compared to the other groups (median AIH ≥ 1/160 (range 1/80–≥ 1/160) versus DI‐ALH 1/160 (range 1/80–1/160) versus DILI 1/80 (range 1/40–≥ 1/160), *p* < 0.001). Positivity for aSMA in the DI‐ALH‐ and DILI‐group was comparable (31% vs. 33%, *p* = 0.659). Positivity for aLKM was comparable in the AIH‐ (14%) and DI‐ALH‐group (19%) whereas all patients in the DILI‐group were negative for aLKM. aSLA was negative in every DI‐ALH case and showed low and comparable positivity in the AIH‐ (6%) and the DILI‐group (9%, Figure S2A).

When comparing patients with immunosuppression for remission induction and those without immunosuppressive therapy, a positive autoantibody serology (any ANA, aSMA, aLKM or aSLA, according to the given definition) could be seen in 97% of the patients with therapy and in 70% in the cases without therapy (*p* < 0.001). Positivity for ANA was 81% in the therapy group compared to 55% in the group without therapy (*p* = 0.012, Figure S2B). ANA titers were comparable in both groups (median immunosuppressive treatment ≥ 1/160 (median 1/80–≥ 1/160) versus no immunosuppressive therapy 1/160 (range 1/80–≥ 1/160), *p* = 0.085). Positivity for aSMA was significantly higher in the treatment group (70% vs. 33%, *p* = 0.002). Median aSMA titre was ≥ 1/160 (range 1/80–≥ 1/160) in the treatment and 1/80 (range 1/40–≥ 1/160) in the no treatment group. aLKM was negative in every case without immunosuppression and positivity for aSLA was comparable (5% vs. 9%, *p* = 0.628, Figure S2B).

### 3.5 Overall Diagnostic Fidelity of pIgG and the Autoantibodies

PIgG had the highest AUC (0.818) to distinguish immune mediated liver diseases (AIH and DI‐ALH) from other liver diseases. AUCs for ANA (0.672), aSMA (0.672), and aLKM (0.576) were comparable and lower for aSLA (0.403).

**FIGURE 2:**
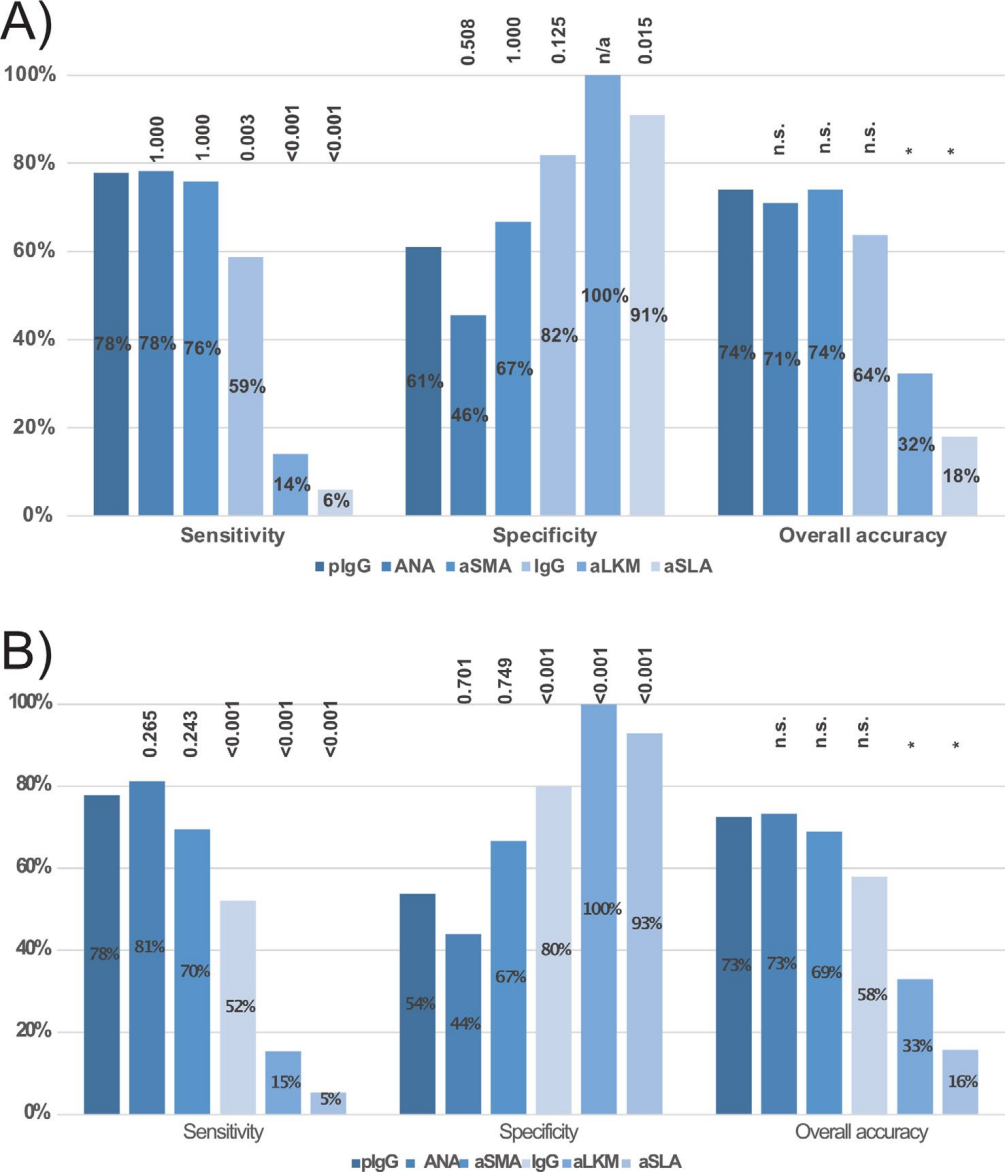
Overall diagnostic fidelity of pIgG, IgG and conventional autoantibodies. Diagnostic fidelity to distinguish untreated AIH from DILI (A) and to distinguish patients with immunosuppressive therapy for remission induction from those without therapy (B). *p*‐values were calculated with the McNemar test for the comparisons of sensitivities and specificities of conventional autoantibodies and IgG in relation to pIgG. * significant difference of accuracies in comparison to pIgG determined by non‐overlapping 95% confidence intervals. aLKM, Anti‐liver kidney microsomal antibodies; ANA, Antinuclear antibodies; aSLA, Anti‐soluble liver antigen antibodies; aSMA, Anti‐smooth muscle antigen antibodies; IgG, Immunoglobulin G; PIgG, Polyreactive immunoglobulin G.

As published recently [13] a cut off of 1.27 nAU for positivity of pIgG and a cut off ≥ 1/40 for positivity in conventional autoantibody measurements via IFT were used to evaluate the diagnostic fidelity of pIgG in comparison to conventional autoantibody measurements in this study. When comparing AIH to DILI with these cut‐offs, sensitivity of pIgG (78%), ANA (78%, *p* = 1.000) and aSMA (76%, *p* = 1.000) were comparable, but significantly lower for IgG (59%, *p* = 0.003), aLKM (14%, *p* < 0.001) and aSLA (6%, *p* < 0.001, Figure 2A). Specificity of aLKM (100%) and aSLA (91%, *p* = 0.015) were higher compared to pIgG (61%). The lowest specificity was seen for ANA (46%, *p* = 0.508 vs. pIgG). Overall accuracy was highest for pIgG and aSMA (both 74%) and comparable to ANA (71%). Overall accuracy was 32% for aLKM and 18% for aSLA.

We hypothesized that the identification of new and higher cut‐offs might increase the accuracy for the distinction of AIH and DILI. Using AUROC analysis and Youden's index, no higher cut‐off improved the overall accuracy of the analysed autoantibodies including pIgG (Table S2A).

When comparing the diagnostic fidelity of pIgG and the conventional autoantibodies to distinguish between patients that needed immunosuppression for remission induction and patients without immunosuppressive therapy, sensitivity of pIgG (cut off of 1.27 nAU, 78%) was not significantly different to ANA (81%, *p* = 0.265) and aSMA (70%, *p* = 0.243, Figure 2B and Table S2B). Sensitivity was lowest for aLKM (15%, *p* < 0.001) and aSLA (5%, *p* < 0.001). Serum IgG had a lower sensitivity than pIgG (52%, *p* < 0.001). Specificity was comparable for pIgG (54%), ANA (44%, *p* = 0.701) and aSMA (67%, *p* = 0.749). Highest specificity was reached for aLKM (100%, *p* < 0.001) and aSLA (93%, *p* < 0.001). IgG had a specificity of 80% (*p* < 0.001). Overall accuracy for pIgG (73%), ANA (73%), aSMA (69%) and serum IgG (58%) were comparable and significantly lower for aLKM (33%) and aSLA (16%).

Again, optimization of cut‐offs by AUROC analysis and Youden Index did not improve overall accuracy (Suppl. Table 2B).

## 4 Discussion

Diagnosing unclear hepatitis can be challenging because immune‐mediated liver diseases with a need for immunosuppressive therapy for remission induction and self‐limiting liver diseases often share similar clinical characteristics at baseline. Testing for autoantibodies using the current gold standard of IFT [16] and liver histopathology is time‐consuming. There is a need for a non‐invasive, cheap, quickly, and widely available biomarker to distinguish these entities and to support decision making for or against immunosuppressive therapy [17].

In 2022, we proposed pIgG as a new diagnostic biomarker for AIH demonstrating higher specificity and greater overall accuracy compared to conventional autoantibodies in a population with a low pretest probability for AIH as typically resembled by real‐world clinical practice [13]. Reactivity in a HIP1R/BSA ELISA was outlined as a surrogate marker for pIgG concentration. In this study, DILI were underrepresented and DI‐ALH was not yet defined, DI‐ALH may have been masked under the label AIH, and a retrospective distinction was not possible. Consequently, the aim of the present study was to include more clearly defined cases of DILI and DI‐ALH in addition to a cohort of AIH, non‐AIH‐non‐DILI‐LD, and healthy controls.

In summary, pIgG was significantly elevated in AIH and DI‐ALH compared to DILI and non‐AIH‐non‐DILI‐LD and the AUC was highest for pIgG. Elevation of pIgG was comparable between both immune‐mediated liver diseases; however, at baseline and during the work‐up of an unclear hepatitis, it is more important to get guidance regarding the need to start immunosuppression to induce remission rather than having the exact discrimination between AIH and DI‐ALH. The finding that pIgG levels are significantly increased in those liver injuries in which the treating physicians saw a need to start immunosuppression is even more important.

The initial analyses were made with the pIgG cut‐off for the distinction between AIH and non‐AIH‐LD from our previous study (1.27 nAU). As for many diagnostic tests, the specificity of the conventional autoantibodies for the diagnosis of AIH could be improved by higher thresholds of the antibody titers, as shown in detail most recently [18, 19]. So, we also tried to identify a higher and more specific pIgG cut‐off for the distinction of AIH and DILI and for liver injuries with/without the need for immunosuppression. This modified higher cut‐off for the positivity of pIgG leads to a higher specificity but at the cost of a lower sensitivity and overall accuracy. The same improvement of specificity with less sensitivity and less overall accuracy could be observed for the conventional autoantibodies as well [18, 19].

While pIgG had a superior accuracy for the distinction between AIH and many different non‐AIH‐liver diseases in adults and children [13, 14], pIgG exhibited comparable accuracies like ANA and aSMA but still higher accuracies than aLKM and aSLA (Figure 2) for the concrete distinction between AIH and DILI or between liver injuries with and without the need for immunosuppression. Interestingly, the pIgG assay exhibited a similar accuracy in the present study (AIH vs. DILI 74%; with/without need for immunosuppression 73%) as in the previous study (AIH vs. non‐AIH liver diseases 73%) (13). However, ANA and aSMA exhibited a better overall performance in the current study (AIH vs. DILI 71% and 74%) compared to the previous study (AIH vs. non‐AIH liver diseases 65% and 64%). The non‐inferiority is not caused by a worse performance of the pIgG assay but by a better performance of the IFT in the present study highlighting again the heterogeneity of performance of IFT across various settings [18, 19]. In addition, pIgG positivity was as high as 79% in patients with AIH being negative for different conventional autoantibodies as well as those with normal IgG thereby complementing current diagnostic standards. What is more, pIgG was especially positive in patients with DI‐ALH caused by drugs that also caused DILI in our cohort. Although sample numbers were low and further large scale studies are needed to validate our findings, pIgG could help to guide decisions in favour of immunosuppressive therapy in this challenging clinical scenario whereas IgG was not able to do so which reflects recent findings questioning the role of IgG in the management of patients with AIH [20, 21, 22].

Current markers are already good in the discrimination between immune‐mediated liver diseases and DILI. Compared to the current gold standard of conventional autoantibody testing via IFT on rodent tissue sections or HEp‐2 cells [3, 16], pIgG are quantified via a solid phase assay with recombinant peptide being less labor‐intensive. Aside from that, the assay can be automated easily and is more objective than IFT, which is dependent on the examiner. Additionally, there is only one dilution step (1/100) in the pIgG assay and not a titration (1/40, 1/80, 1/160 etc.), which leads to less serum demand, which is especially relevant in children.

Limitations of this retrospective study are small sample numbers for DILI and DI‐ALH due to the analysis of rare diseases and available biomaterial was even rarer limiting the possibility to associate presence of pIgG for example, with longitudinal clinical disease course. Additionally, important quality measures were implemented such as a mandatory liver biopsy for AIH and DI‐ALH and clinical follow‐up regarding the short‐ and long‐term dependency on immunosuppression in case of DILI and DI‐ALH. The liver biopsies were reviewed in a decentralised manner at each participating center, without a standardised assessment. Retrospective studies are limited by incomplete data. For example, some patients could not remember details about their medical therapy, which limited for example, the adjudication to specific substances in cases of DILI and DI‐ALH. In addition, due to retrospective data collection, the type and dosage of immunosuppressive therapy in patients with AIH at 6 months was unavailable for some patients with their usage of immunosuppression being only retrievable in a dichotomous manner. The current cohort also has the bias of non‐standardised autoantibody measurement. We have just initiated a prospective multicenter study to validate the accuracy of pIgG for the prediction of AIH, DI‐ALH, DILI and other non‐viral liver diseases as well as the need for immunosuppression in liver injuries (NCT05810480). We expect the first results in 2028 to 2029.

To summarise the findings of previous studies [13, 14, 15, 19] and the present study, the evaluation of pIgG via the HIP1R/BSA assay demonstrated higher diagnostic precision for pIgGs in cases of low pretest probability of AIH when compared to other liver diseases, excluding those specifically enriched for DILI or DI‐ALH. This enhanced precision can be attributed primarily to the elevated specificity of pIgG in comparison to conventional autoantibodies. However, in the challenging subgroup analysis of AIH, DILI, and DI‐ALH with a high pretest probability for AIH (AIH *n* = 81 vs. DILI *n* = 23 or DI‐ALH *n* = 16) with the need to decide for or against steroid use (steroids *n* = 94 vs. no steroid use *n* = 23), pIgG is not inferior to conventional autoantibodies.

## 5 Conclusion

We conclude that pIgG has the potential to serve as a promising supplementary biomarker, with the capacity to differentiate between immune‐mediated liver diseases and DILI, thereby addressing the existing limitations in current diagnostic methodologies. The potential for its utilisation in the prediction of an immunosuppression‐dependent liver injury is comparable to that of conventional autoantibodies ANA and aSMA in IFT, albeit with a less labor‐intensive ELISA. A prospective evaluation of pIgG has been initiated in Europe; however, the results remain to be published.

## Author Contributions

Study concept and design: Richard Taubert, Bastian Engel. Acquisition of data: Theresa Kirchner, Richard Taubert, Bastian Engel, George N. Dalekos, Kalliopi Zachou, Mercedes Robles‐Díaz, Raúl J. Andrade, Marcial Sebode, Ansgar Lohse, Maciej K. Janik, Piotr Milkiewicz, Mirjam Kolev, Tony Bruns, Nasser Semmo, Sarah Habes, Ye H. Oo, Claudine Lalanne, Simon Pape, Joost P. H. Drenth, Luigi Muratori, Jessica K. Dyson, Isabel Graupera, Benedetta Terziroli Beretta‐Piccoli. Drafting of the manuscript: Theresa Kirchner, Richard Taubert, Bastian Engel. Critical revision of the manuscript for important intellectual content: George N. Dalekos, Kalliopi Zachou, Mercedes Robles‐Díaz, Raúl J. Andrade, Marcial Sebode, Ansgar Lohse, Maciej K. Janik, Piotr Milkiewicz, Mirjam Kolev, Tony Bruns, Tom Jg Gevers, María‐Carlota Londoño, Sarah Habes, Ye H. Oo, Claudine Lalanne, Simon Pape, Joost P. H. Drenth, Luigi Muratori, Jessica K. Dyson, Isabel Graupera, Benedetta Terziroli Beretta‐Piccoli, Heiner Wedemeyer, Elmar Jaeckel. Statistical analysis: Theresa Kirchner, Richard Taubert, Bastian Engel. Obtained funding: Richard Taubert, Bastian Engel. Administrative, technical, or material support: Richard Taubert, Bastian Engel, Heiner Wedemeyer, Elmar Jaeckel. Study supervision: Richard Taubert, Bastian Engel.

## Funding

Bastian Engel was supported by the PRACTIS—Clinician Scientist Programme of Hannover Medical School, funded by the German Research Foundation (DFG, ME 3696/3) and by a bridging program as part of the CORE100Pilot for clinician scientists in transplantation medicine, funded by Else Kröner Fresenius Foundation (2020_EKSP.78) and the Ministry for Science and Culture of Lower Saxony (ZN3720). B.E. and R.T. were supported by the ForTra gGmbH für Forschungstransfer der Else Kröner‐Fresenius‐Stiftung (ForTra) (2024_EKTP01 to B.E. and R.T.).

## Disclosure

Richard Taubert and Elmar Jaeckel are inventors of the patent application for the use of anti‐HIP1R/BSA for the diagnosis of AIH (European patent: EP 3701264 B1; US patent: US 12044682 B2). EUROIMMUN Medizinische Labordiagnostika AG and Inova Diagnostics Inc. provided ELISAs free of charge for other projects of Richard Taubert and Bastian Engel. Ye H. Oo, collaborator from the pIgG study group, thanks the Sir Jules Thorn Charitable Trust and Whitney Wood Fellowship. All other authors have nothing to disclose with regard to this paper.

## Conflicts of Interest

The authors declare no conflicts of interest.

## Supporting information


**Data S1:** liv70571‐sup‐0001‐Supinfo.docx.

## Data Availability

The data that support the plots within this paper and other findings of this study are available from the corresponding authors upon reasonable request.
