# Supplementary material for: Elevated Polyreactive Immunoglobulin G in Immune‐Mediated Liver Injuries With the Need for Immunosuppressive Therapy
Source: Liver Int. 2026 Mar 8;46(4):e70571. doi: 10.1111/liv.70571 (PMC12968488; doi:10.1111/liv.70571)
Supplement: Supplementary file 1 — Data S1: liv70571‐sup‐0001‐Supinfo.docx. [file LIV-46-0-s001.docx]

**Elevated polyreactive immunoglobulin G in immune-mediated liver injuries with the need for immunosuppressive therapy**

Theresa Kirchner^1, 11^, George N. Dalekos²^, 11^, Kalliopi Zachou²^, 11^, Mercedes Robles-Díaz³, Raúl J. Andrade³, Marcial Sebode^4, 11^, Ansgar Lohse^4, 11^, Maciej K. Janik^5, 11^, Piotr Milkiewicz^5,6, 11^, Mirjam Kolev^7^, Nasser Semmo^7^ ,Tony Bruns^8, 11^, [Tom Jg. Gevers](https://pubmed.ncbi.nlm.nih.gov/?term=Gevers+TJ&cauthor_id=36440259)^9, 11^, [Benedetta Terziroli Beretta-Piccoli](https://pubmed.ncbi.nlm.nih.gov/?term=Terziroli+Beretta-Piccoli+B&cauthor_id=36440259)^10^, Heiner Wedemeyer^1, 11^, Elmar Jaeckel^1, 11, +^, ^#^Richard Taubert^1, 11^, ^#^Bastian Engel^1, 11, 12^and pIgG study group.

^1^ Department of Gastroenterology, Hepatology, Infectious Diseases and Endocrinology, Hannover Medical School, Hannover, Germany.

² Department of Medicine and Research Laboratory of Internal Medicine, National Expertise Center of Greece in Autoimmune Liver Diseases, General University Hospital of Larissa, Larissa, Greece.

³ Unidad de Gestión Clínica de Aparato Digestivo, Instituto de Investigación Biomédica de Málaga-IBIMA, Plataforma BIONAND, CIBEREHD, Hospital Universitario Virgen de la Victoria, Universidad de Málaga, Málaga, Spain.

^4^ Department of Medicine, University Medical Centre Hamburg-Eppendorf, Hamburg, Germany.

^5^ Department of Hepatology, Transplantology, Internal Medicine, Medical University of Warsaw, Warsaw, Poland.

^6^ Translational Medicine Group, Pomeranian Medical University, Szczecin, Poland

^7^ Department of Visceral Surgery and Medicine, Inselspital, Bern University Hospital, University of Bern, Bern, Switzerland.

^8^ Department of Medicine III, University Hospital RWTH Aachen, Aachen, Germany.

^9^ Department of Gastroenterology and Hepatology, Maastricht University Medical Center, Maastricht, Netherlands.

^10^ Epatocentro Ticino, Lugano, Switzerland.

^11^ European Reference Network on Hepatological Diseases (ERN RARE-LIVER).

+ current address: Ajmera Transplant Center, Toronto General Hospital, United Health Network, University of Toronto, Toronto, Canada.

^#^ shared last authorship

**Correspondence:** Dr. Bastian Engel & Prof. Dr. Richard Taubert; Hannover Medical School; Dept. Gastroenterology, Hepatology, Infectious Diseases and Endocrinology; Carl-Neuberg-Straße 1; 30625 Hannover; Germany. Tel.: +49-511-532-6766, Taubert.Richard@mh-hannover.de & [Engel.Bastian@mh-hannover.de](mailto:Engel.Bastian@mh-hannover.de)

**Table of content**

Title page 1

Supplementary figures 2

Supplementary tables 5

Supplementary methods 8

References 9

**Supplementary Figures**

**Supplementary Figure 1**

**A)**

**B)**

**C)**

**D)**

**E)**

**Supplementary Figure 1 – Positivity of polyreactive IgG in cases with negativity for conventional autoantibodies and/or immunoglobulin G**

Frequencies of positive (blue) and negative (orange) pIgG tests in patients negative for antinuclear antibodies (ANA), anti-smooth muscle antigen antibodies (aSMA), anti-liver kidney microsomal antibodies (aLKM), anti-soluble liver antigen antibodies (aSLA) and normal immunoglobulin G (IgG) in AIH (A), DI-ALH (B), DILI (C), patients that were treated with immunosuppressive therapy (D) and patients that did not need immunosuppressive treatment (E).

**Supplementary Figure 2**

**A)**

**B)**

**Distribution of antibody- and immunoglobulin G test results across groups**

Positivity and titer for conventional autoantibodies and IgG in the different groups (A) and in patients with and without immunosuppressive therapy for remission induction (B). Abbrevations: pIgG: polyreactive immunoglobulin G, ANA: antinuclear antibodies, aSMA: anti-smooth muscle antigen antibodies, aLKM: anti-liver kidney microsomal antibodies, aSLA: anti-soluble liver antigen antibodies, IgG: immunoglobulin G, AIH: autoimmune hepatitis, DI-ALH: drug-induced autoimmune-like hepatitis, DILI: drug-induced liver injury.

**Supplementary tables**

**Suppl. Table 1: Immunosuppressive therapy**

|  | | **AIH (n=81)** | **DI-ALH (n=16)** | **DILI (n=23)** | **p** |
| --- | --- | --- | --- | --- | --- |
|  |  | median (min; max) / n (%) | median (min; max) / n (%) | median (min; max) / n (%) |  |
| **Simplified AIH Score^§^** | | 7 (6; 9) | 5 (4; 5) | 5 (2; 7) | <.001 |
| **Immunosuppressive treatment for remission induction** | **yes** | 79 (98) | 15 (94) | 0 (0) | <.001 |
| **Immunosuppressive drug for remission induction** | **steroids** | 40 (51) | 15 (100) |  | .018 |
|  | **steroids+MMF** | 22 (28) | 0 (0) |  |  |
|  | **steroids+azathioprine** | 9 (11) | 0 (0) |  |  |
|  | **steroids+azathioprine+MMF** | 1 (1) | 0 (0) |  |  |
|  | **other** | 7 (9) | 0 (0) |  |  |
| **Immunosuppressive treatment at month 6** | **yes** | 79 (98) | 0 (0) | 0 (0) | <.001 |
| **Immunosuppressive drug at month 6** | **steroids** | 27 (34) |  |  |  |
|  | **MMF** | 4 (5) |  |  |  |
|  | **steroids+MMF** | 17 (22) |  |  |  |
|  | **steroids+azathioprine** | 18 (23) |  |  |  |
|  | **other** | 3 (4) |  |  |  |
|  | **Not specified** | 10 (13) |  |  |  |
| ^§^ Hennes et al, Simplified criteria for the diagnosis of autoimmune hepatitis. Hepatology. 2008  Combination of medication were prescribed sequentially.  MMF: mycophenolate mofetil. | | | | | |

**Suppl. Table 2: Diagnostic fidelity for AIH vs DILI (2A) and immunosuppressive therapy vs no immunosuppressive therapy (2B) for pIgG, conventional autoantibodies at modified cut-offs**

**2A**

|  | Sensitivity | 95% CI | Specificity | 95% CI | Overall accuracy | 95% CI |
| --- | --- | --- | --- | --- | --- | --- |
| pIgG cut off 1.71 nAU | 0.556 | 0.447-0.659 | 0.913 | 0.732-0.985 | 0.635 | 0.525-0.789 |
| ANA cut off ≥1/160 | 0.689 | 0.564-0.790 | 0.583 | 0.320-0.807 | 0.671 | 0.492-0.718 |
| aSMA cut off 1/160 | 0.695 | 0.569-0.800 | 0.857 | 0.497-0.993 | 0.712 | 0.660-0.834 |

**2B**

|  | Sensitivity | 95% CI | Specificity | 95% CI | Overall accuracy | 95% CI |
| --- | --- | --- | --- | --- | --- | --- |
| pIgG cut off 2.00 nAU | 0.413 | 0.352-0.572 | 1 | 0.852-1 | 0.643 | 0.456-0.813 |
| ANA cut off ≥1/160 | 0.714 | 0.659-0.842 | 0.675 | 0.591-0.835 | 0.551 | 0.425-0.732 |
| aSMA cut off 1/160 | 0.576 | 0.482-0.743 | 0.913 | 0.799-0.943 | 0.439 | 0.391-0.678 |

ANA: antinuclear antibodies, aSMA: anti-smooth muscle antigen antibodies, pIgG: polyreactive immunoglobulin G, 95% CI: 95% confidence interval.

**Suppl. Methods**

**Quantification of polyreactive immunoglobulin G**

Patients’ serum samples from Hannover Medical School were cryo-conserved at below -20°C. Serum samples from external centers were cryo-conserved according to local protocols and sent frozen to Hannover Medical School for centralized quantification of pIgG. Samples were pseudonymized for autoantibody-testing and observers were blinded to any clinical information.

Quantification of pIgG using an ELISA to quantify reactivity to a peptide and BSA as blocking agent was performed as published^1^. In short, 0.01 µg of HIP1R fragment per well was bound to 96 well ELISA plates over night at 4 °C. Plates were blocked with TBS and 5 % BSA for 30 minutes. Plates were washed with TBS with Tween20® 0.05 % (TBST) once. Serum samples were diluted 1:101 (v/v) in TBS and 5 % BSA and 100 µl per well were added to the ELISA plate and incubated for two hours. Plates were washed three times with TBST and incubated with a secondary rabbit anti-human anti-IgG antibody labeled with horseradish peroxidase for 30 minutes. Three washing steps with TBST were performed and 3, 3', 5, 5' tetramethyl benzidine (BioLegend, San Diego California) was added for 30 minutes for color reaction. Reaction was stopped with sulfuric acid. Optical density was read at 450 nm using an ELISA reader (Tecan Sunrise-Basic, Grödig, Austria). Sera of five patients with gradual increase in pIgG reactivity were measured in every experiment and used to compute a standard curve. Arbitrary units (AU) were calculated from the equation of the standard curve. Measurements were performed in Hannover, Germany.

As different AU dependent on center and storage duration were demonstrated, a normalization for these factors (referred to as normalized AU (nAU)) was performed as published^1^.

**Immunofluorescence testing**

IFT was performed by experienced technicians using the recommended methodology of the guidelines issued in 2004 by the Committee for Autoimmune Serology of the International Autoimmune Hepatitis Group^2^. Samples were pseudonymized for autoantibody-testing and observers were blinded to any clinical information. ANA, anti-SMA, anti-LKM and anti-LC1 were detected by IFT on sections of frozen rodent liver, stomach and kidney sections. Briefly, a commercial rodent multi-organ substrate panel (kidney, liver and stomach) was used (LKS Rat wrapped Standard Kit, Aesku.Diagnostics GmbG & Co. Wendelsheim, Germany). The sera were diluted, starting with a dilution of 1:20 up to 1:320, and applied to the slide to cover the entire tissue section and allow binding of the autoantibodies to the substrates. After washing, the sample was exposed to a second fluorochrome-labeled antibody. Finally, once washed again, the slides were examined under fluorescence microscope (Olympus BX60 Microscope, Evident Europe GmbH, Germany), and the antibody staining pattern was evaluated and interpreted accordingly to the guidelines^2^.

# **References**

1. Taubert R, Engel B, Diestelhorst J, et al. Quantification of polyreactive immunoglobulin G facilitates the diagnosis of autoimmune hepatitis. *Hepatology*. 2022;75(1):13-27. doi:10.1002/hep.32134

2. Vergani D, Alvarez F, Bianchi FB, et al. Liver autoimmune serology: a consensus statement from the committee for autoimmune serology of the International Autoimmune Hepatitis Group. *J Hepatol*. 2004;41(4):677-683. doi:10.1016/j.jhep.2004.08.002
